# BC-PROM: validation of a patient-reported outcomes measure for patients with breast cancer

**DOI:** 10.1097/MD.0000000000006781

**Published:** 2017-04-28

**Authors:** Xiaojuan Hu, Chunsen Zhang, Yanbo Zhang

**Affiliations:** From the Department of Health Statistics, School of Public Health, Shanxi Medical University, Taiyuan, Shanxi, China.

**Keywords:** breast cancer, instrument development, item response theory, patient-reported outcomes, psychometrics

## Abstract

Key issues of treatment for patients with breast cancer such as patient satisfaction with treatments, compliance, and some side effects are essential for evaluating quality of life by patient-reported outcomes in clinical trials. The study aimed to develop and evaluate a specific patient-reported measure which included physical, psychological, social, and therapeutic domain for assessing the survival of patients with breast cancer.

The pool of items was drafted after a theoretical revision and cognitive interviews with women with breast cancer. The draft scale was formed after the adjustment of the items and dimensions, and the selected items were submitted to expert's judgments. Five statistical methods were used to select these items by 2 validation samples. The final scale was administered to a sample of 417 patients from 8 hospitals and 135 controls for reliability, validity, and responsive analyses.

The final BC-PROM consisted of 52 items, 13 subdomains, and 4 domains, being developed after preexamination and formal examination. Cronbach alpha coefficient was 0.902 and 0.712 for the full scale and therapeutic domain. The structural validity results showed that the multidimensional measurement of the scale fulfilled expectations. Differences in the BC-PROM mean scores were significant between cancer patients and healthy participants in 13 subdomains (*P* < .05), indicating good responsiveness. Among the sample survey of patients, the scale copy acceptance rate was 98.2%, completion rate 94.6%, and average filling time 10 minutes.

The new and reliable BC-PROM was developed in patients with breast cancer and applied to clinical treatment evaluation and clinical trials for such patients.

## Abstract 2

Supplemental Digital Content is available in the text

## 1 Introduction

Breast cancer is one of the most important female cancers in many countries, and its incidence has increased in recent decades.^[1]^ That rise is attributed to the more widespread use of mammography and a consequent earlier detection of the disease. Earlier detection—and therefore earlier treatment—is expected to result in improved survival rates.^[2]^ The improvement in survival rates has been related to the concern with quality of life (QOL) among the surviving breast cancer patients.^[3]^ With advanced treatment, the role of the patient shifts to that of survivor, and there is a need for continued focus on overall QOL issues.^[4]^

Generally, the evaluating indices of QOL mainly include 2 aspects: information from physician and patient-rated indices. There is some difference between physician and patient-rated indices in survival status of patient with breast cancer. The patient rating is viewed as more useful because it includes subjective information only patients can provide. There is a paradigm shift in evaluating the outcomes of medical care in the past years. The outcome assessment has a more focus on the patients’ perception of their health than clinical indexes of disease activity. The term “patient-reported outcome” (PRO) has been used to denote the inclusion of the patient's viewpoint in medical care.^[5]^ The US Food and Drug Administration (FDA) 2006 draft guidance “Patient-Reported Outcome Measures: Use in Medical Product Development to Support Labeling Claims” has engendered wide discussion about PRO domains that should be taken as the end points in clinical trials.^[6]^ PRO refers to outcomes that arise directly from patients’ perceptions of their own health conditions.^[7]^ In the case of cancer, PRO is used to assess the overall burden of the disease and the effectiveness of interventions.

There has been increased the use of outcome measures in both clinical practice and research to capture data regarding a patient's self-reported level of disability and determine the relative effectiveness of interventions.^[8]^ The European Organization for Research and Treatment of Cancer-Breast Module and Functional Assessment of Cancer Therapy-Breast (FACT-B) were widely translated and used by many countries to measure QOL in breast cancer population.^[9]^

Due to the number of items and domains, scoring, and psychometric properties, there are great differences in these instruments. These items of QOL are more important in breast cancer survivors. Apart from some clinical symptoms, patients with breast cancer experience a series of nonclinical symptoms. Feelings of depression and isolation are mostly experienced. Additionally, patients with breast cancer are so fatigue that they have difficulty in daily life. The research findings have indicated that such factors as fatigue, anxiety, body image, and sexuality strongly affect QOL following breast cancer diagnosis and treatment.^[10]^ Developments in therapeutic interventions and humanistic concerns have transformed the prospects for breast cancer patients. PRO for a patient with a history of breast cancer assess the impacts of disease, treatment, and side effects related to different treatment modalities on various aspects of a patient's outcomes.^[11]^ The interventions of providing social support could improve QOL.^[9]^

Such patient-reported outcomes measure for breast cancer (BC-PROM) includes the following: measures of physical functioning, emotional functioning, social functioning, and therapeutic aspects.^[12]^ The most indexes of patient-reported outcomes measure (PROM) are subjective indicators, and strongly influenced by the regional economic and cultural background in the evaluation of health status. Under the circumstance, there is no recognized and accurate scale of patients with breast cancer. So development and validation of a new specific BC-PROM which suit regional economic and cultural background is indispensable. The information collected from the scale is used to evaluate the effect of cancer therapy, and screening the best choices of treatment and new anticancer drugs in clinical oncology.

## 2 Methods

### 2.1 Ethics statement

The study protocol (No.2013098) received medical and ethical approval from the Shanxi Medical University. We obtained written informed consent from all participants.

### 2.2 Study population

We enrolled patients from 8 different hospitals in Shanxi Province, China. The investigators had studied training manuals and acquired investigative techniques through unified training. All participants were requested to complete the scale independently after receiving a brief introduction about the BC-PROM from one of the investigators. If a patient was unable to complete the questionnaires by themselves, they received assistance from an individual who had a proper understanding of the patient's condition. If the patients or their assistants encountered any problems with the questions, investigators were able to provide a detailed explanation. The questionnaires were filled in so as to reflect the patient's current situation. If the patients or their assistants were illiterate or had a low educational level, an investigator read out the questions and options, though without providing any actual guidance. Depending on the patient's own wishes, they could receive the assistance of an investigator or an assistant in completing the questionnaire.

Prior to the study, the investigators received relevant medical knowledge about general surgery and oncology. Furthermore, the authors made every effort to ensure that the investigators adopted a serious, responsible attitude so as to maintain the quality of this study. To minimize any missing data, the investigators checked the questionnaires immediately after completion.

The inclusion criteria for the breast cancer patients were as follows: having a definite diagnosis of breast cancer; being over 18 years of age; and being willing to undergo the investigation. The exclusion criteria were as follows: a patient with mental illness or disturbance of consciousness; inability to understand or complete the scale owing to deficiencies of language or cognitive ability; and being unwilling to undertake the investigation. Control participants were collected to meet the following criteria: not suffering from breast cancer, cancer, and mental illness; the overall age of healthy people was similar with that of patients with breast cancer; and being a volunteer to join in the research of this subject. Health controls also provided informed consent and got some rewards.

We tested the missing data in the questionnaires using Little missing completely at random test, and set a *P* value of <.001. If the data were being missing at random, we replaced them based on the expectation-maximization algorithm.^[13]^

### 2.3 Sample size

In order to obtain stable and reliable analysis results and accurate parameter estimates, some scholars have suggested that the actual survey data sample should be 5 to 10 times of the observed variables. Nunnally^[14]^ suggested that the number of subjects was at least 10 times that of the study variables in factor analysis. For the first item-selection process, we recruited 149 breast cancer patients; valid data were obtained from 137 participants. We selected 102 patients and 35 controls, who participated in the presurvey. For the 2nd item-selection process and validation of the BC-PROM, we recruited 446 breast cancer patients and 141 controls from the same 8 regions. Of those, 417 patients and 135 controls were able to complete the final scale.

### 2.4 Scale scoring

For each item, patients responded using a 5-point Likert scale to reflect how often they experienced the issues in past2 weeks. We assigned initial values to each category, ranging from 0 to 4. The responses were 0 = never, 1 = occasionally, 2 = about half of the time, 3 = often, and 4 = almost every day. Scores of positively worded items were recoded as the original score plus 1; scores of negatively worded items were recoded as 5 minus the original response. This recoding produced a score range for each item of 1 to , with a higher score reflecting a more positive PROM.

### 2.5 Development and formation of BC-PROM

**Figure 1:**
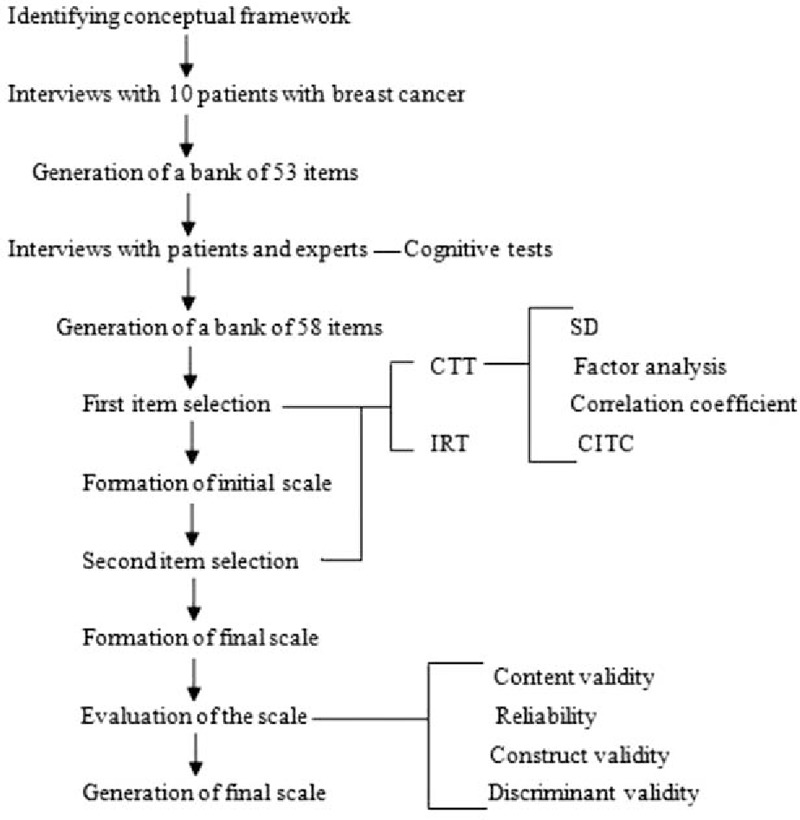
Flowchart of the patient-reported outcomes measures for breast cancer developmental process. CITC = corrected item-total correlation, CTT = classical test theory, IRT = item response theory, SD = standard deviation.

The PROM for breast cancer was developed in 4 phases: conceptual framework construction and preliminary item generation; development of the initial scale by the 1st item-selection process; formation of the final scale based on the 2nd item-selection process; and validation of the PRO measure. A flowchart of this 4-phase developmental process appears in Fig. 1. We mainly used the methods of classical test theory (CTT) and item response theory (IRT) in the 2 item-selection processes.

### 2.6 Conceptual framework construction

Following the principles for PRO measurement tools provided by the FDA, we conducted a literature search of academic databases for PRO instruments and available Net resources for breast cancer. We then formed a conceptual framework for the new instrument. We developed 4 domains and 13 subdomains as follows: physical domain (subdomains: breast symptom, chest symptom, systemic symptom, unique reaction, and independence), psychological domain (subdomains: anxiety and depression, self-abasement, and despair), social domain (subdomains: social support and social adaptation), and therapeutic domain (subdomains: satisfaction, compliance, and drug side effects).

### 2.7 Item generation

We conducted in-depth open-ended interviews of 10 breast cancer patients to identify potential items for the BC-PROM using the selected conceptual framework.^[15]^ Patients were interviewed to discuss their main physical feelings and symptoms, psychological and social burden, and satisfaction with treatment. We recorded the main points of information from those interviews.

We selected 10 patients to take a cognitive test. Those patients were requested to indicate items that they found vague or difficult to understand. Based on the patients’ suggestions, we added or removed some items. As a result, we generated a bank of some potential items. We made revisions to the scale following interviews with these experts, and the preliminary scale was formed.

### 2.8 Statistical analysis

#### 2.8.1 Item selection

We used the methods of CTT and IRT to evaluate the items. An item was considered for selection when it was retained by 4 or more methods. However, the evaluation also included a consideration of each item's practical significance. Together, the statistical results and practical significance of the items contributed to improvements of the scale.^[16]^

#### 2.8.2 Classical test theory (CTT)

We used 4 methods to evaluate the items in the CTT analyses. We assessed the sensitivity of items using the standard deviation (SD): an item was deleted if its standard deviation was ≤1. We employed factor analysis to assess the subdomains. Items with low factor loading (<0.40) and with factor loading close to other factors were deleted. An item was considered for deletion when the Pearson correlation between the item and its superior factor was <0.50. Internal consistency was assessed with Cronbach alpha coefficient and the corrected item-total correlation. An item was considered for deletion when the corrected item-total correlation was <0.50 and the item's deletion increased the Cronbach alpha coefficient.^[17]^

#### 2.8.3 Item response theory (IRT)

IRT models have been the preferred methodology for statistically analyzing survey responses and patients’ latent traits.^[18]^ We assessed items using MultiLog 7.03 with Samejima graded response model to investigate the measurement properties. The graded response model is suitable for analyzing ordered response categories, such as Likert-type rating scales.^[19]^ We used plots of an item's characteristic curves to demonstrate efficiency. Each item was characterized by 2 parameters, namely discrimination parameter and difficulty parameter. We determined these with maximum likelihood estimation. The practical values of the item parameters for deletion were as follows: a < 0.4; b (–3, 3).^[20]^

#### 2.8.4 Validation of final scale

We evaluated the final BC-PROM for validity, reliability, and responsiveness using the data obtained from those 417 patients as well as the 135 control participants.

#### 2.8.5 Reliability

We calculated Cronbach alpha coefficients for 4 domains and the total scale to measure the internal consistency of the BC-PROM. Generally, a value of more than 0.70 indicates that individual items provide an adequate contribution to the overall scale.^[21]^

#### 2.8.6 Validity

Content validity. The relevant literatures and subject patients’ opinions were typically consulted in validating content validity which how well these items met the empirical indexes of interest.^[5]^

Construct validity. We subjected the factor structure of the scale to confirmatory factor analysis (CFA). The model was contrasted for relative goodness-of-fit statistics, such as the following: root mean square error approximation (values < 0.08 indicate adequate fit and values < 0.05 indicate a close fit of the data to the model); ^[22]^ normed fit index (values ≥0.90); nonnormed fit index (values ≥0.90); incremental fit index (values ≥0.90); comparative fit index (values ≥0.90); and root mean square residual (values < 0.09).^[23]^ Using LISREL 8.70, we assessed construct validity with CFA.

Discriminant validity. We determined discriminant validity by comparing the mean scores for every subdomain of the BC-PROM among control participants and the groups of breast cancer patients. We compared the differences using analysis of variance, with the significance level set at *P* < .05.^[21]^

#### 2.8.7 Feasibility

We evaluated the feasibility of the BC-PROM by examining the response rate, completion rate, and response time to completion. We considered response and return rates of less than 95% inadequate and completion times of 8 to 13 minutes acceptable.

## 3 Results

### 3.1 Participant characteristics

In the 1st item-selection phase, 102 from breast cancer patients and 35 from control participants were returned. The subjects of breast cancer were 18 to 68 years old, with an average of 45.12 ± 12.04 years (see Appendix 1, Supplemental Content, which described demographic characteristic of patients and controls in the 1st item-selection phase). In the 2nd item-selection phase, 417 breast cancer patients and 135 controls agreed to participate. The subjects of breast cancer were 20 to 73 years old, with an average age of 47.97 ± 10.31 years. We calculated and compared demographic information using the Z-test for continuous variables and chi-square test for categorical variables (see Appendix 2, Supplemental Content, which described demographic characteristic of patients and controls in the 2nd item-selection phase).

### 3.2 Generation of item pool and matrix plot of item characteristic curves

**Figure 2:**
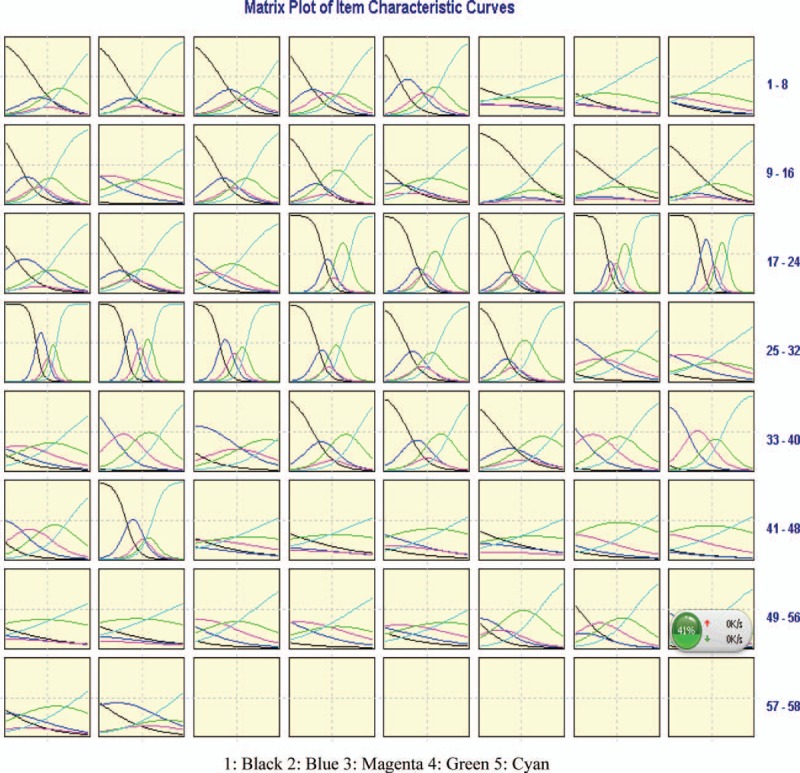
Item-characteristic curves and information functions using the graded-response model. The colored curves are item-characteristic curves, each corresponding to a different response category; the dashed lines are item information functions. Five curves from left to right correspond to options 1 to 5.

We undertook a comprehensive review to form 4 domains, 13 subdomains, and 53 potential items. Then we made an interview with some experts to revise the various fields of the scale. We finally created a preliminary scale of 58 items. The matrix plot of item characteristic curves (ICCs) with IRT for each item is showed in Fig. 2. Each item of the BC-PROM had 5 response categories. For each item in ICCs, each response category was represented by 1 curve. Ideally, the 1st and last curve of ICCs should be monotonic for each item. And the remaining 3 curves (ie, curves of blue, magenta, and green) should be approximately normal distribution. As shown in Fig. 2, the ICCs were closer to the ideal case for a majority of items.

### 3.3 Item selection

#### 3.3.1 The 1st item-selection phase

In our study, the Kaiser–Meyer–Olkin statistic was 0.706, which was larger than the gold criteria of Kaiser–Meyer–Olkin (0.7). Meanwhile the *P* value was <.001 in Bartlett test of sphericity. These 2 statistics indicated the factor analysis was right for the data. The cumulative variance contribution rate of the first 13 factors was up to 75.145%. Therefore, we selected 13 factors and assigned each item to the corresponding factors. For CTT and IRT in the 1st item-selection phase, the selection and some statistical results of the items appear in Table 1. The items PHD10, PSD15, SOD3, SOD10, and SOD12 should have been removed in the results. However, many patients and experts agreed that there is a need to ask and investigate with regard to item SOD12 (Do you take an active part in beneficial social activities?), and so we decided to retain it. Therefore, we deleted 4 items from the initial scale. The scale was left with 4 domains, 13 subdomains, and 54 items.

**Table 1:**
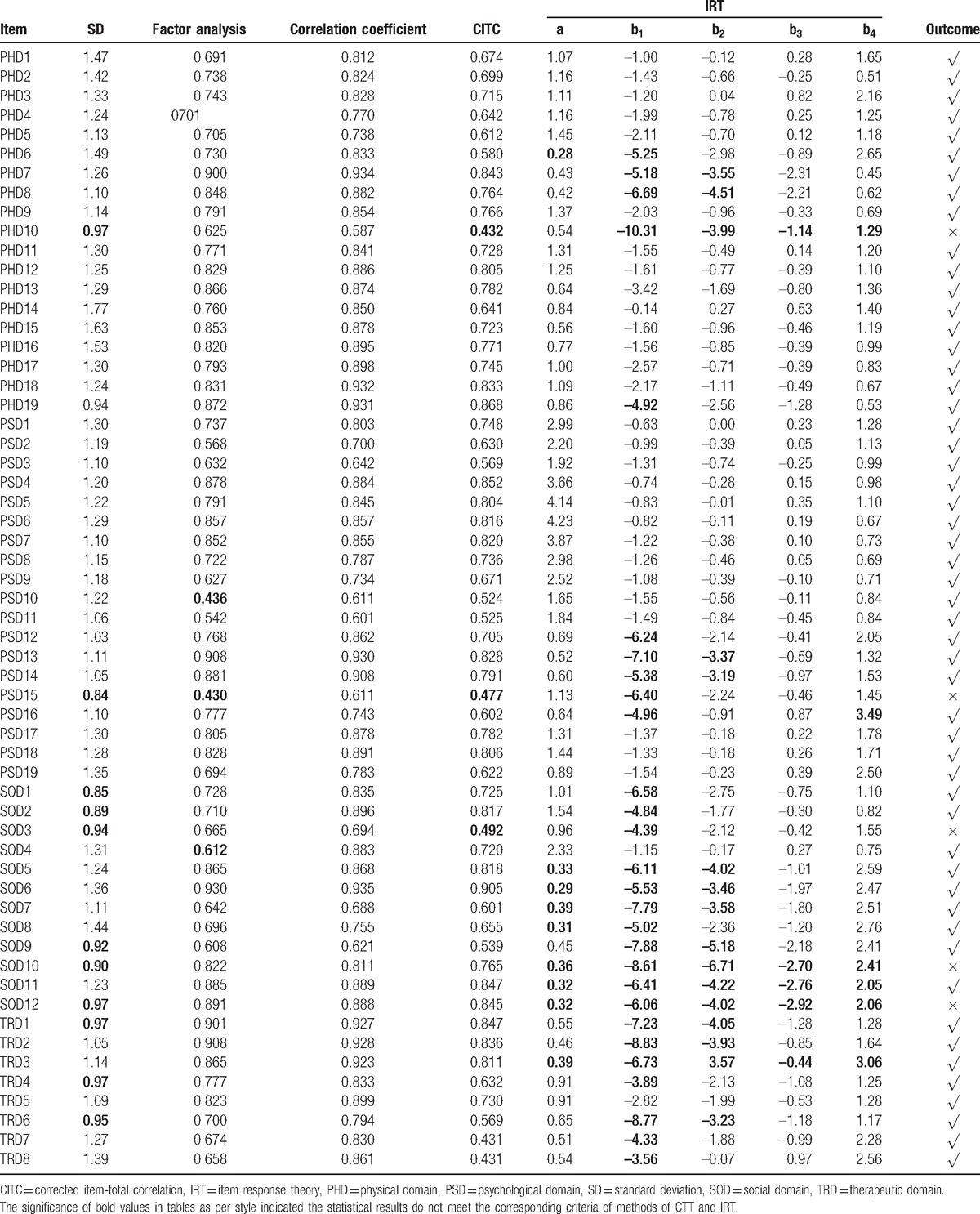
Summary of the 1st item-selection phase using classical test theory and item response theory.

#### 3.3.2 The 2nd item-selection phase

With CTT and IRT in the 2nd item-selection phase, the selection and some statistical results of the items appear in Table 2. Items PSD10 (Have you changed your understanding and pursue of life?) and PSD15 (Are you worried about that you will die?) had to be removed according to the above criteria. Therefore, 2 items were deleted from the final scale, which consisted of 4 domains, 13 subdomains, and 52 items (see Table 3).

**Table 2:**
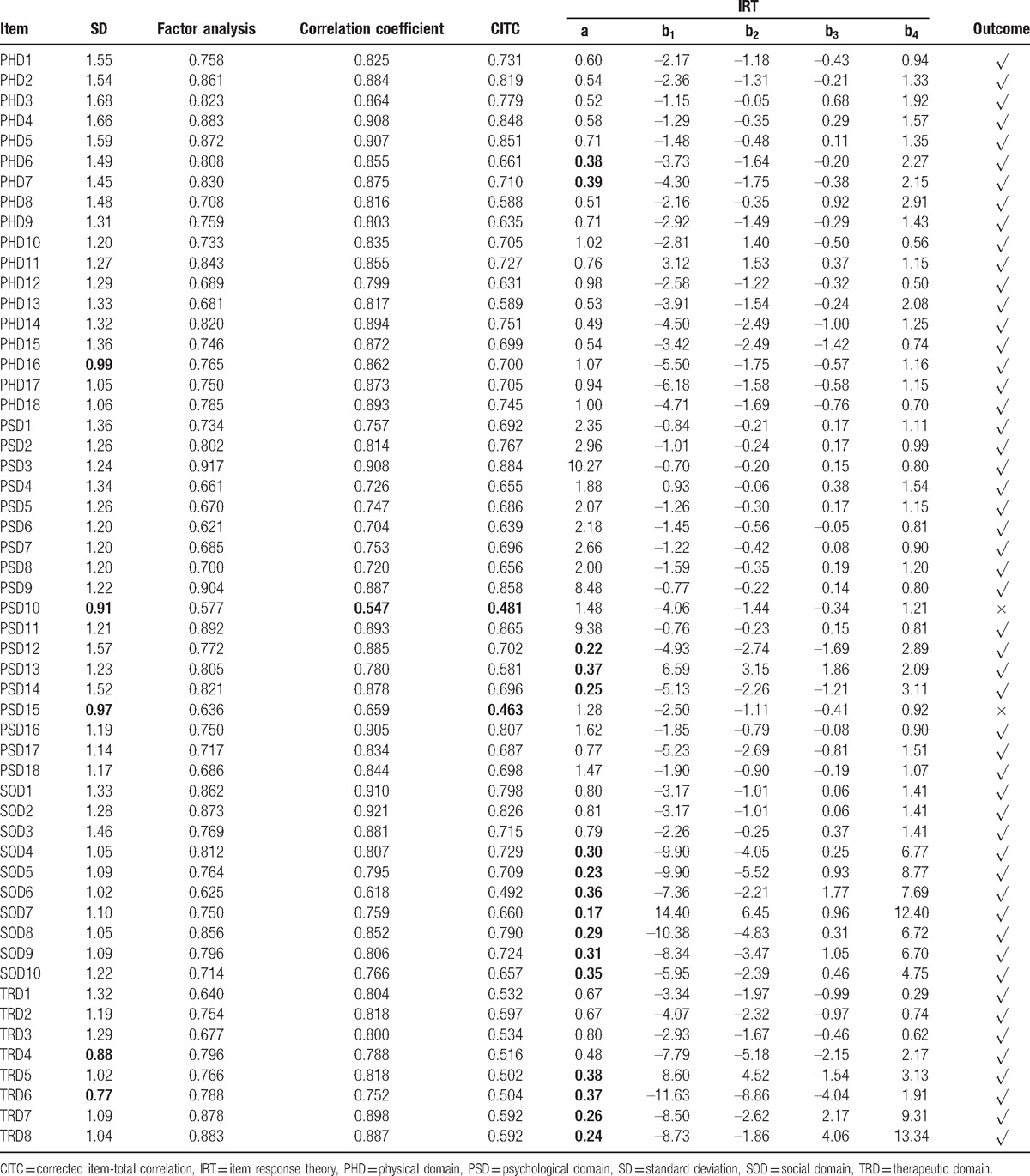
Summary of the 2nd item-selection phase using classical test theory and item response theory.

**Table 3:**
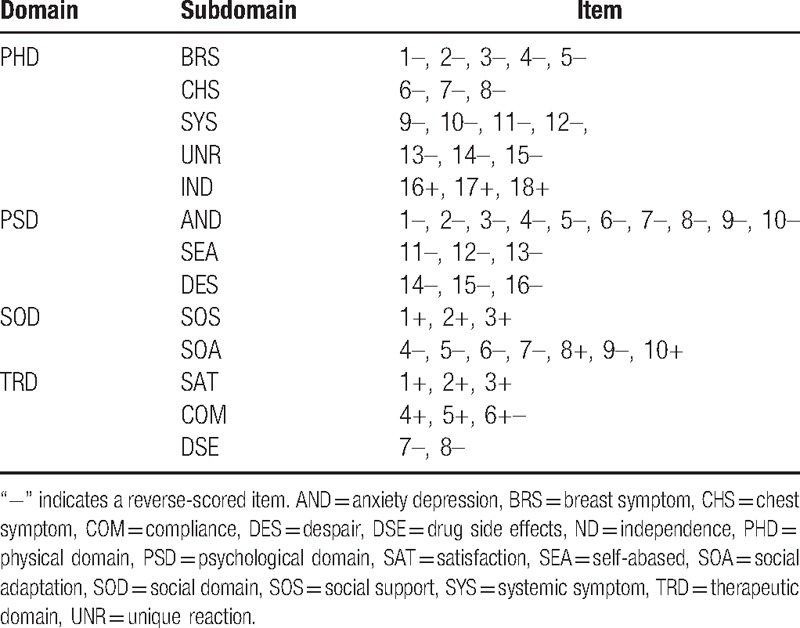
Scale structure of the bank of 52 items of the final scale.

#### 3.3.3 Validation of the scale

Additional analyses focused on examining the reliability and validity of the BC-PROM (ie, 52 items, 4 domains) using classical measurement techniques.

#### 3.3.4 Reliability

Cronbach alpha coefficients for the 4 domains and overall scale appear in Table 4. As evident in that table, Cronbach alpha coefficients are 0.867, 0.884, 0.826, 0.712, and 0.902 for physical domain, psychological domain, social domain, therapeutic domain, and total scale, respectively. The 52-item BC-PROM demonstrated a high degree of internal consistency reliability.

**Table 4:**
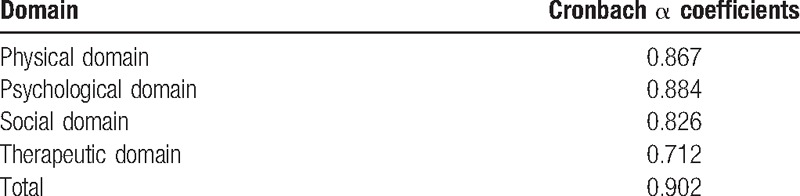
Reliability of the 4 domains and the whole scale.

#### 3.3.5 Content validity

We achieved content validity by referring to the relevant literature. To ensure that all the items were appropriate and relevant, we consulted questionnaires from China and other countries. We also interviewed 10 patients to identify potential items; we consulted with 5 patients, 3 physician experts, and 1 psychometric expert for item revision and refinement.

#### 3.3.6 Construct validity

The indexes of fit for 4 domains are mostly presented in following. Indicators of model fitness used include the root-mean-square error approximation (0.079 for PHD, 0.13 for PSD, 0.079 for SOD, and 0.007 for TRD), normed fit index (0.94 for PHD, 0.92 for PSD, 0.96 for SOD, and 0.97 for TRD), nonnormed fit index (0.95 for PHD, 0.92 for PSD, 0.96 for SOD, and 0.98 for TRD), the comparative fit index (0.96 for PHD, 0.93 for PSD, 0.97 for SOD, and 0.99 for TRD), and incremental fit index (0.96 for PHD, 0.93 for PSD, 0.97 for SOD, and 0.99 for TRD). The fit statistics met the defined criteria, which was strongly suggested by the high factor loadings. The results of CFA appear in Table 5. The standardized factor loadings for most of the items were >0.5; construct validity was therefore deemed satisfactory.

**Table 5:**
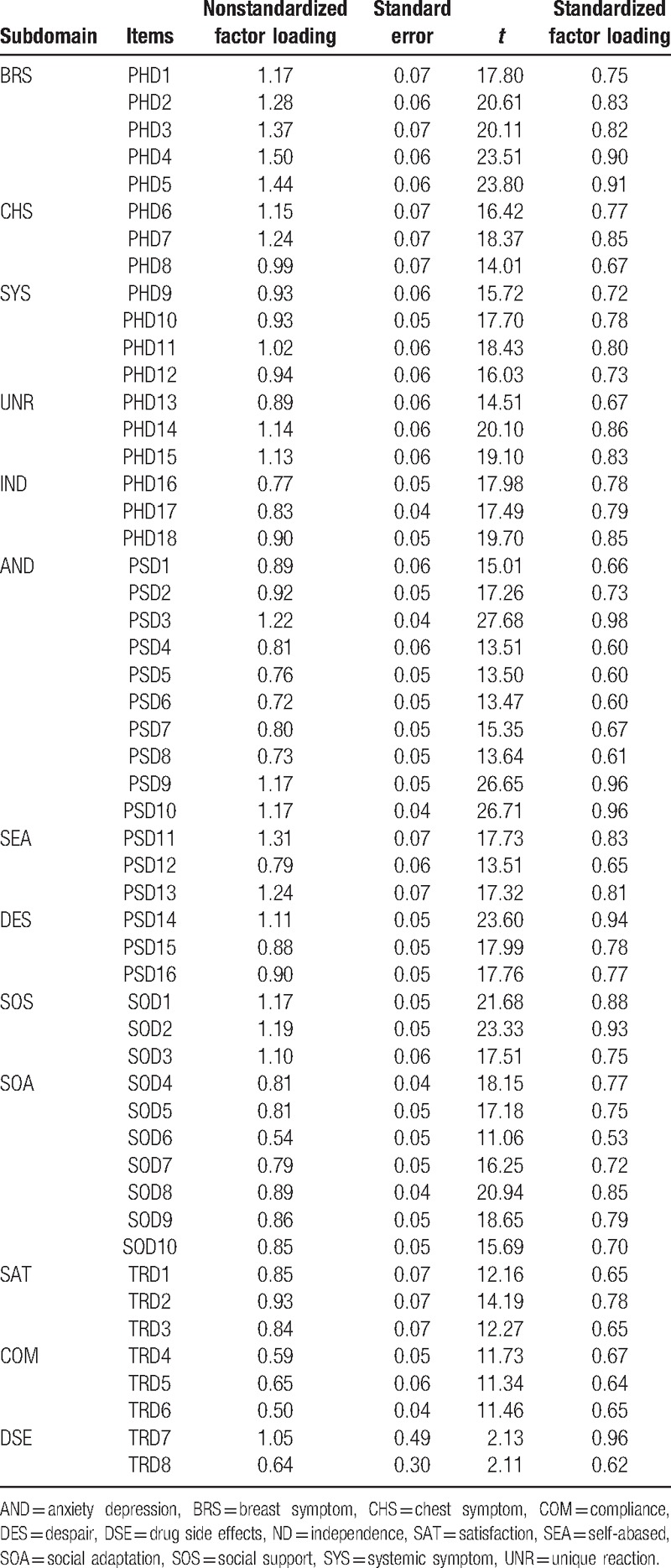
Results of the confirmatory factor analysis.

#### 3.3.7 Discriminant validity

As shown in Table 6, the discriminant validity of each subdomain was examined by comparing mean scores in the 2nd validation samples (417 patients, 135 controls). Based on *t* tests, the rejection of the null hypothesis of each subdomain indicated that the scale had the ability to differentiate between controls and patients.

**Table 6:**
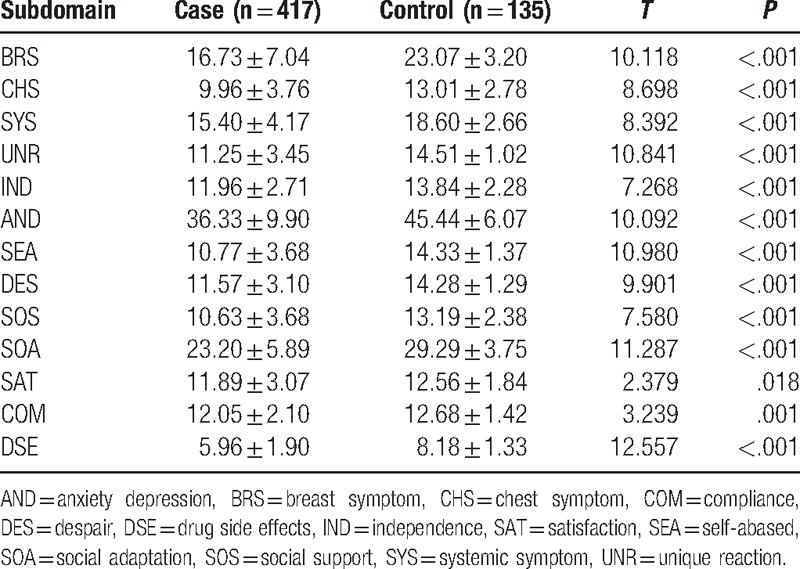
Scores comparisons between control participants and patients with breast cancer.

#### 3.3.8 Feasibility

The response rate of the BC-PROM was 90.31%. The effective rate of return of the scale was 94.04%. The majority of participants were able to complete the scale within 10 minutes.

## 4 Discussion

With the number of women with breast cancer increasing annually among many countries, breast cancer is one of the leading causes of death in women. In such an environment, it is really essential to get more acquainted with the information of one's health-related QOL.^[24]^ Currently, more and more patients are involved in evaluations of health-care quality using PRO.^[21]^ The purpose of this study was to establish a reliable and valid patient-reported scale for assessing the QOL of breast cancer patients. The BC-PROM which had validated by 2 samples from 8 hospitals included areas of QOL and broader concepts, such as patient satisfaction with care and some side effects. The results of our study indicated that the BC-PROM is a valid instrument for measuring survival state for women with breast cancer.

We evaluated 4 aspects of our scale: reliability, validity, responsiveness, and feasibility. To decrease the study burden and funding, we did not evaluate test–retest reliability. We used Cronbach alpha coefficient to examine reliability. We employed the CFA method to assess construct validity. Responsiveness was evidenced by the score differences between controls and breast cancer patients. Finally, we evaluated feasibility on the basis of the effective rate of return and the time required to complete the scale. The Cronbach alpha coefficient for the breast subscale was 0.59 in Wan study on the Chinese version of FACT-B scale.^[25]^ But the findings of this study show that the reliability is appropriate. Cronbach alpha reliability coefficient for total scale is 0.902.

During the item-selection phase, we combined CTT and IRT. CTT is easy to implement in the software of SPSS. Cronbach alpha of 4 subdomains and the whole scale was calculated which described the reliability of BC-PROM. However, CTT was item-sample dependent,^[26]^ and its application was restricted in the development and validation of the scale. The analyses based on IRT can increase the accuracy and efficiency of the BC-PROM. Results through IRT provided much richer information on the performance of each item.^[27]^ It is useful during the development or refinement of our scale. ICCs provided through IRT reflected the accuracy of measurement at different values of the latent trait.^[28]^ By examining the probability of endorsing response categories for each item, it ensured that the best items are remained. Graded response theory was one of several models in IRT. It was used on the characteristics of ordered polytomous categories in our paper.

Many existing instruments measuring QOL have undergone development and validation among patients.^[12]^ Referring to the pros and cons of those currently available PRO instruments, we developed a PROM to assess the survival of breast cancer patients. This study examined measurement properties, such as the reliability, validity, and responsiveness of the instrument. Finally, we developed a scale that consisted of 4 domains and 13 subdomains as follows: physiology, psychology, society, and treatment. The helpful information gathered by this instrument is used to be as prognostic and medical factors. Studies of BC-PROM can further indicate the directions needed for more efficient treatment of cancer patients. The scientific evidence from QOL data can be used to assist in clinical decision making during the phase of diagnosis and treatment for breast cancer.^[29]^

The BC-PROM covered more symptoms and treatment-related side effects than the Functional Assessment of Cancer Therapy–Breast Symptom Index. In the present study, the questionnaires were validated in the breast cancer population of community health centers in 3 Chinese cities. However, owing to the difficulties in following up patients and the desire not to impose an excessive burden on patients, we did not measure the test–retest reliability in the validation process of the BC-PROM. Thus, internal consistency was used only to illustrate the reliability of this scale. However, we did conduct reliability evaluation of the items with respect to both item selection and evaluation of the scale.

In 1st item-selection phase of this study, the sample size was less than 5-fold the number of selected items, and so factor analysis could not be used to explore the 4 subdomains. We need to explore this problem in future studies.

The study participants were female breast cancer patients. Because of the special symptoms of this disease, the items we assessed were related to the physiological characteristics of women. There are thus limits to the application of our scale. Owing to limited funds and other resources, our study population was restricted to Shanxi Province in northern China. Further development and wider application of the BC-PROM should be validated using nationwide or non-Chinese samples in future studies.^[12]^

## 5 Conclusion

This study makes an important contribution to the treatment of breast cancer and the wider health-measurement fields. We used mixed methods (CCT, IRT, and CFA) to identify beneficial items in the BC-PROM, which may be widely applied in other health areas. The validated BC-PROM could be applied to evaluate clinical treatment and clinical trials of new medicines for breast cancer patients.

### 5.1 Other Information

All authors declared that the work described was original research that has not been published previously, and not under consideration for publication elsewhere, in whole or in part. The manuscript had been read and approved by the authors, that the requirements for authorship as stated in the Uniform Requirements for Manuscripts Submitted to Biomedical Journals had been met.

## Acknowledgements

The authors thank the cooperation of the community hospitals at Taiyuan City, Linfen City and Jincheng City. The authors also thank the National Natural Science Foundation of China [81273180] for the support.

## Supplementary Material

Supplemental Digital Content
