# Supplementary material for: BC-PROM: validation of a patient-reported outcomes measure for patients with breast cancer
Source: Medicine (Baltimore). 2017 Apr 28;96(17):e6781. doi: 10.1097/MD.0000000000006781 (PMC5413279; doi:10.1097/MD.0000000000006781)
Supplement: Supplemental Digital Content [file medi-96-e6781-s001.doc]

Appendix 1 Demographic characteristic of 102 patients with breast cancer and 35 controls in the

**first item-selection phase**

| Characteristics | Patients($n$=102) | Controls($n$=35) | t或Z | $\text{p}$ |
| --- | --- | --- | --- | --- |
| Age（mean,SD,years） | 45.12(12.04) | 47.54(11.18) | -1.047 | 0.297 |
| Height (mean,SD,cm) | 162.60(7.30) | 161.51(6.33) | 0.783 | 0.435 |
| Weight (mean,SD,kg) | 54.69(7.09) | 55.63(7.20) | -0.676 | 0.500 |
| Age at menarche(N) |  |  |  |  |
| ≤13 | 30 | 7 | 1.313 | 0.519 |
| 14-16 | 54 | 20 |  |  |
| ≥17 | 18 | 8 |  |  |
| Age at menopause(N) | (*n*=74) | (*n*=28) |  |  |
| ≤44 | 12 | 7 | 4.752 | 0.191 |
| 45-55 | 41 | 18 |  |  |
| ≥56 | 21 | 3 |  |  |
| Age of first full-term pregnancy(N) | (*n*=92) | (*n*=32) |  |  |
| ＜25 | 23 | 5 | 1.396 | 0.706 |
| 25-29 | 56 | 21 |  |  |
| ≥30 | 13 | 6 |  |  |
| No. of children(N) |  |  |  |  |
| 0 | 10 | 3 | 0.330 | 0.848 |
| 1-2 | 41 | 16 |  |  |
| ＞2 | 51 | 16 |  |  |
| Abortion times(N) |  |  |  |  |
| 0 | 30 | 19 | 7.257 | 0.027 |
| 1-2 | 37 | 7 |  |  |
| ＞2 | 35 | 9 |  |  |

Appendix 2 Demographic characteristics of 417 patients with breast cancer and 135 controls in the

**second item-selection phase**

| Characteristics | Patients($n$=417) | Controls($n$=135) | t或Z | $\text{p}$ |
| --- | --- | --- | --- | --- |
| Age | 47.97(10.31) | 47.61(11.09) | 0.350 | 0.727 |
| Height (cm) | 162.14(6.48) | 161.73(7.17) | 0.634 | 0.526 |
| Weight (kg) | 54.31(7.05) | 55.63(7.21) | -1.736 | 0.083 |
| Menarche age |  |  |  |  |
| ≤13 | 121 | 32 | 1.580 | 0.454 |
| 14-16 | 204 | 73 |  |  |
| ≥17 | 92 | 30 |  |  |
| Menopause age | (*n*=338) | (*n*=116) |  |  |
| ≤44 | 54 | 20 | 5.579 | 0.134 |
| 45-55 | 189 | 74 |  |  |
| ≥56 | 95 | 22 |  |  |
| Age of first delivery | (*n*=405) | (*n*=127) |  |  |
| ＜25 | 97 | 25 | 3.758 | 0.289 |
| 25-29 | 244 | 82 |  |  |
| ≥30 | 64 | 20 |  |  |
| Number of births |  |  |  |  |
| 0 | 12 | 8 | 3.627 | 0.163 |
| 1-2 | 125 | 45 |  |  |
| ＞2 | 280 | 82 |  |  |
| Abortion times |  |  |  |  |
| 0 | 200 | 74 | 1.932 | 0.381 |
| 1-2 | 123 | 34 |  |  |
| ＞2 | 94 | 27 |  |  |
